# Capitellum fractures: should the collateral ligament be repaired primarily?

**DOI:** 10.3389/fsurg.2025.1597973

**Published:** 2025-07-16

**Authors:** Zonghuan Li, Aixi Yu, Weijuan Zeng

**Affiliations:** ^1^ Department of Orthopedics Trauma and Microsurgery, Zhongnan Hospital of Wuhan University, Wuhan, Hubei, China; ^2^ Department of Ophthalmology, Zhongnan Hospital of Wuhan University, Wuhan, Hubei, China

**Keywords:** capitellum fracture, collateral ligament injury, systematic review, review, ligament repair

## Abstract

**Background:**

It is controversial whether the collateral ligaments should be repaired primarily for capitellum fractures with ligamentous injury. This research was conducted to summarize the current evidence for this issue.

**Methods:**

Databases, including Medline, Cochrane library and EMBASE, were searched from their establishment to December 31, 2024 for clinical articles on capitellum fractures. The reference lists of the relevant studies were also checked successively. The general information including first author, publication time, location, the number of cases, treatment for the capitellum fractures with collateral ligament injury, were included. Outcomes, including the pronation and supination of the elbow, active range of motion, Mayo elbow performance score, elbow instability and complications, were extracted.

**Results:**

Fifteen studies and 220 patients were identified and analyzed. The average follow-up time ranged from 1.5 to 17 years. The fractures were managed by open reduction and internal fixation. Medial collateral ligaments (MCL) injury was reported in 4 of the 15 included reports. Among the 97 patients, 17 (17.5%) patients suffered capitellum fracture with MCL injury. For the treatment of MCL injury, the literature reports were inconsistent. Nine studies with 159 patients reported the treatment for the lateral collateral ligaments (LCL) injury. Fifty-six cases were complicated with LCL injury, of which 41 cases were primary injury and 15 LCL were released to enhance exposure. All LCL injuries were repaired primarily.

**Conclusion:**

For capitellum fractures with ligament injuries, primary repair of LCL should be performed when combined by LCL injury or LUCL release is performed during surgery. The MCL may require primary reconstruction or treatment in a hinged brace.

## 1 Introduction

Capitellum fracture is a rare elbow fracture, accounting for about 1% of elbow fractures (1, 2). Capitellum fractures typically occur when an individual falls on an outstretched hand with the forearm in pronation, transmitting force through the radial head to impact the capitellum (3). It often causes coronal fracture of distal humerus. The fracture block moves upward and even turns over 180 degrees. When the force reaches a sufficient magnitude, it can be combined with the injury of radial head, posterior condyle of distal humerus, lateral epicondyle, olecranon of ulna, medial and lateral collateral ligament injuries (4). For displaced capitellum fractures, surgical treatment is the optimal method, including open reduction and internal fixation, fracture block resection, joint replacement and so on (5, 6). However, it is still controversial whether the collateral ligaments should be repaired in one stage for capitellum fracture with ligament injury. Some people insist that one-stage repair should be performed to reconstruct the anatomical structure and restore the stable fixation of the elbow (7–9). Nevertheless, literature shows that ligament injury is mostly caused by elbow instantaneous valgus, and complete rupture is rare (10). From this point of view, primary repair is generally not recommended, and one-stage repair will increase the risk of complications such as incision infection, ulnar nerve injury, and postoperative heterotopic ossification leading to elbow stiffness (10).

The purpose of this study is to conduct a systematic review of the current literature on whether or not to perform one-stage ligament repair for capitellum fracture with ligament injuries.

## 2 Methods

### 2.1 Search strategy

Databases including Medline, Cochrane library and EMBASE were systematically searched for clinical research on capitellum fractures from their establishment to December 31, 2024. Considering the limited number of clinical studies related to capitellum fractures with collateral ligament injury, we included all studies related to capitellum fractures, regardless of whether they were associated with ligament or other injuries. Then the full text was reviewed to screen and include those on capitellar fractures with ligament injuries. Ligament injuries include the following two situations: (1) Ligament injuries caused by trauma, including medial collateral ligament (MCL) or lateral collateral ligament (LCL) injuries; (2) release of the lateral ulnar collateral ligament (LUCL) required to enhance exposure during surgery.

Medical Subject Headings together with the free words (“capitellum”, “capitulum”, “capitellar”, “coronal shear fracture”) were used. The reference lists were also checked for additional studies successively.

The search strategies were as the following:

Medline: ((capitellum[Title/Abstract]) OR (capitulum[Title/Abstract]) OR (capitellar [Title/Abstract]) OR (coronal shear fracture[Title/Abstract])).

Cochrane library: capitellum in Title Abstract Keyword OR capitulum in Title Abstract Keyword OR capitellar in Title Abstract Keyword OR coronal shear fracture in Title Abstract Keyword.

EMBASE: (“capitellum”/exp OR capitellum OR “capitulum”/exp OR capitulum OR capitellar OR “coronal shear fracture”/exp OR “coronal shear fracture” OR ((“coronal”/exp OR coronal) AND (“shear”/exp OR shear) AND (“fracture”/exp OR fracture))) AND ([cochrane review]/lim OR [controlled clinical trial]/lim OR [systematic review]/lim OR [randomized controlled trial]/lim OR [meta analysis]/lim).

Two authors independently screened the titles and abstracts to identify potentially relevant studies. Full text of all identified studies was obtained and then reviewed. Studies meeting the inclusion and exclusion criteria were selected.

### 2.2 Inclusion and exclusion criteria

The inclusion and exclusion criteria were constructed as the following.

Inclusion criteria: (i) patients diagnosed as capitellum fractures; (ii) patients were surgically treated; (iii) one or more outcome(s) [Mayo elbow performance score (MEPS), pronation and supination, active range of motion (ROM), pain, elbow instability, complications] was (were) described; (iv) No restrictions were placed on study design. No language restriction was set.

Exclusion criteria: (i) distal humeral fracture without involving capitellum; (ii) case report; (iii) review, course, experimental research or technique introduction; (iv) repetitive study.

### 2.3 Data extraction

Data extraction of all included studies was performed independently by two authors. The general information (first author, published year, country/region, cases, gender, age, cases with ligament injury, intervention, approach, postoperative treatment, follow-up time) were extracted. All outcomes and related complications as mentioned above were extracted for systematic review.

### 2.4 Quality assessment

The risk of bias (ROB) tool provided by Cochrane collaboration was adopted to evaluate the methodological quality of included randomized controlled trials (RCTs) (11). The ROB tool consists of 7 items including random sequence generation, allocation concealment, blinding of participants and personnel, blinding of outcome assessment, incomplete outcome data, selective reporting and other bias. Each item can be evaluated as “low risk”, “unclear risk” and “high risk”.

### 2.5 Data analysis

Statistical analysis was conducted with software RevMan (version 5.3). Mean difference (MD) and relative risk (RR), both with 95% confidence intervals (CI), were used to analyze continuous and dichotomous data, respectively. A *P* value <0.05 was considered statistically significant. Narrative synthesis was performed when comparative data were not available.

## 3 Results

### 3.1 General description of included literature

**Figure 1:**
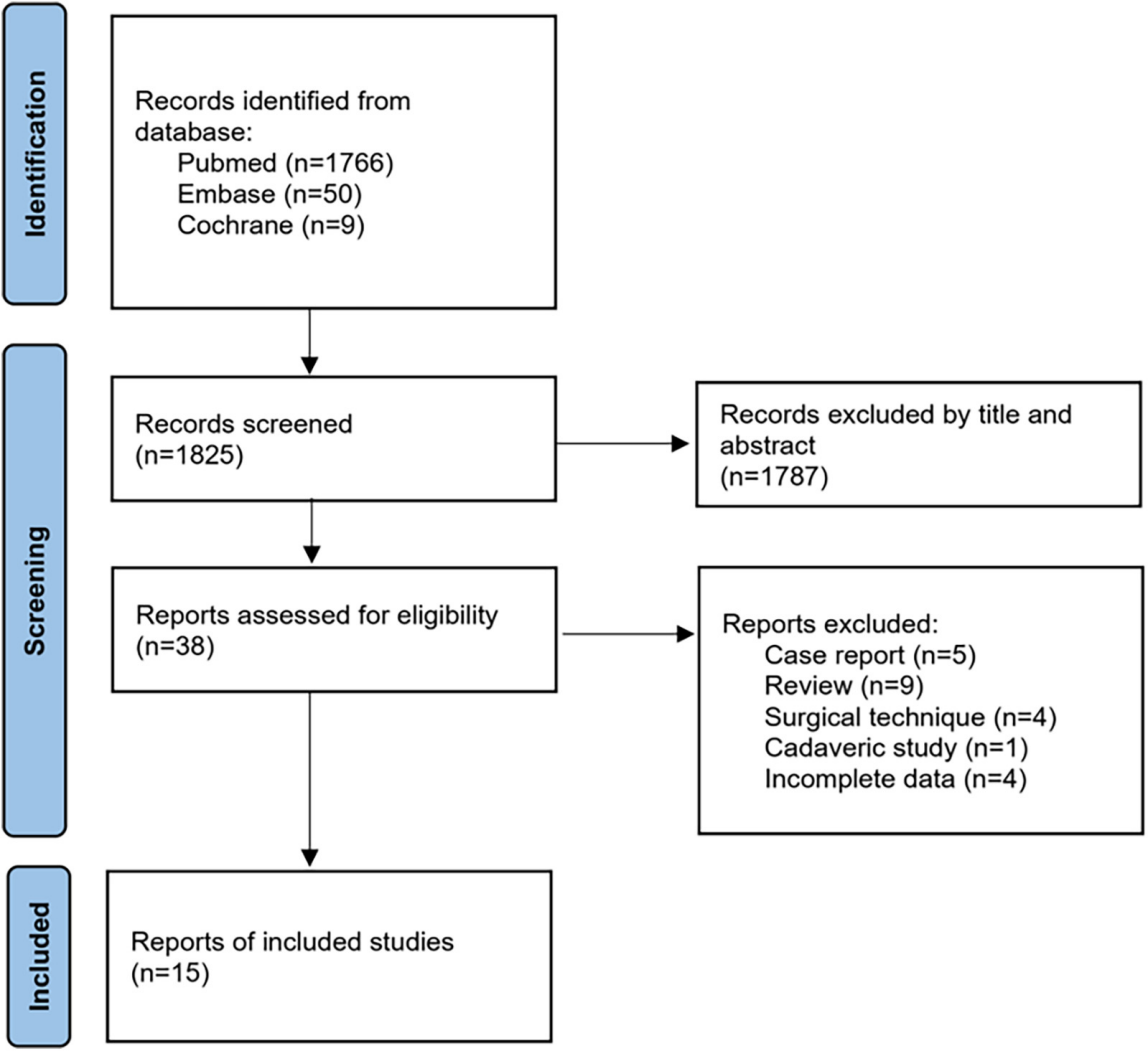
The literature retrieval and screening flowchart according to the PRISMA.

The literature retrieval and screening flowchart is shown in Figure 1. A total of 1,825 (Medline 1,766, Embase 50, Cochrane 9) studies were obtained from the database search and reference list check. Thirty-eight studies remained after examination by screening the title and abstract. Full-text of these studies were retrieved and checked strictly. Case reports (5), reviews (9), surgical technique (4), cadaveric study (1) and studies with incomplete data (4) were excluded successively. Finally, 15 studies with 220 patients were included in our analysis (7, 12–25).

### 3.2 Characteristics of included literature

The general information was listed in Table 1. All included studies were retrospective or prospective reports without controlled group. Thus, the quality assessment of the methodology was not performed. Most patients were young and middle-aged, with an average age range from 26 to 62. The publication year ranged from 1991 to 2020. Among the 220 patients, 59 were male and 136 were female, while the rest were not reported. The average follow-up time ranged from 1.5 to 17 years. The fractures were managed by open reduction and internal fixation, while the treatment for the associated injury varied. The severity of ligament injuries and their surgical indications were listed in Table 2. As stated in the table, most ligament ruptures were confirmed during intraoperative exploration. The ruptured ligaments were repaired after fracture fixation. If instability still persisted, external fixation was applied as adjuvant treatment.

**Table 1:** General information of the included studies.

Study	Location	Cases	Gender (m/f)	Age (years)	Follow-up time	Approach
Giannicola et al. (14)	Rome, Italy	15	5/10	47 (18–65)	29 (12–49) months	Lateral Kocher approach (12), posterior midline incision (3)
Zhang et al. (10)	Beijing, China	28	18/10	34 (14–66)	28.5 (12–72) months	Kocher approach (16), lateral approach combined with a medial approach (1), anterior elbow approach (2), posterior median approach (9)
Dubberley et al. (13)	Ontario, Canada	28	4/24	43 (20–71)	56 (14–121) months	Posterior midline skin incision (24), lateral skin incision (4)
Chang et al. (12)	New York, UK	9	-	26–70	1.5 years	Olecranon osteotomy
Ashwood et al. (7)	Staffordshire, UK	26	13/13	39.4 (22–76)	46 (19–94) months	Kocher interval (10), medial side of the elbow (3), olecranon osteotomy (13)
Singh et al. (22)	Delhi, India	14	9/5	33 (16–46)	4.8 (4–7) years	Kocher interval
Ruchelsman et al. (20)	New York, UK	16	—	40 ± 17	27 ± 19 months	Extensile lateral exposure
Guitton etal. (15)	Amsterdam, Netherlands	14	6/8	29 (20–41)	17 (7–23) years	No standard protocols (lateral skin incision, posterior incision, olecranon osteotomy)
Mighell et al. (19)	Florida, USA	16	3/13	not report	13 (7–24) months	Lateral approach
Sano et al. (21)	Chiba, Japan	6	0/6	51 (12–78)	5.6 (2.5–9.3) years	Lateral approach (4), posterior approach with olecranon osteotomy (2)
Imatani et al. (16)	Okayama, Japan	6	1/5	47 (38–66)	40 (24–54) months	Anterolateral approach
McKee et al. (18)	Boston, USA	5	1/5	38 (10–63)	22 (18–26) months	Lateral approach
Liberman et al. (17)	Beer-Sheva, Israel	5	2/3	26 (13–40)	—	Lateral approach
Tarallo et al. (24)	Modena, Italy	24	—	50.2 (18–71)	30 (24–40) months	Extensile lateral Kocher approach
Tarallo et al. (23)	Modena, Italy	8	6/2	50 (37–64)	30 (12–40) months	Posterolateral Kocher approach

**Table 2:** The severity of ligament injuries and their surgical indications.

Study	Ligament injury	Surgical indication
Giannicola et al. (14)	Not mentioned	Not mentioned
Zhang et al. (10)	Not mentioned	When intraoperative confirmation
Dubberley et al. (13)	Medial collateral ligament tear intraoperatively hinged brace)	Not mentioned
Chang et al. (12)	Not mentioned	Not mentioned
Ashwood et al. (7)	Diagnosed intraoperatively	Evidence of collateral ligament instability
Singh et al. (22)	Diagnosed under general anesthesia by manipulation under fluoroscopy	Elbow instability after ORIF
Ruchelsman et al. (20)	Diagnosed intraoperatively	When intraoperative confirmation
Guitton et al. (15)	Not mentioned	Not mentioned
Mighell et al. (19)	Posterior subluxation of the radial head	When intraoperative confirmation
Sano et al. (21)	Not mentioned	Not mentioned
Imatani et al. (16)	Not mentioned	Not mentioned
McKee et al. (18)	Not mentioned	Not mentioned
Liberman et al. (17)	Not mentioned	Not mentioned
Tarallo et al. (24)	Identified intraoperatively	When the fragment with the ligament attached was displaced
Tarallo et al. (23)	Identified intraoperatively	The lateral collateral ligament, when injured, was reinserted to its humeral origin with transosseous sutures.

### 3.3 Incidence of capitellum fracture with ligament injury

MCL injury was reported in 4 of the 15 included reports (Table 3). Among the 97 patients, 17 (17.5%) patients suffered capitellum fracture with MCL injury. For the treatment of MCL injury, the literature reports were inconsistent. Nine studies with 159 patients reported the treatment for the LCL injury (Table 4). Fifty-six cases were complicated with LCL injury, of which 41 cases were primary injury and 15 LUCL were released to enhance exposure. All LCL injuries were repaired primarily.

**Table 3:** Treatment and outcomes for capitellum fracture with MCL injury.

Study	Cases with MCL injury	Intervention	Pronation and supination	Active ROM	MEPS	EI	Complication
Giannicola et al. (14)	9/15	Hinged elbow fixator	Full	13°–140°	98 (75–100)	1	Pain (1)
Zhang et al. (10)	2/28	Hinged elbow fixator	—	112°(60°–150°)	92.5 (62–100)	0	None
Dubberley et al. (13)	2/28	Hinged brace (1), ligament repair (1)	Loss of 4°	19°–138°	91 (65–100)	—	None
Ashwood et al. (7)	4/26	Repaired with suture anchors	Loss of over 10°	14.1°–128.8°	81.3 (65–100)	0	Pain (2)

ROM, range of motion; MEPS, Mayo elbow performance score; EI, elbow instability.

**Table 4:** Treatment and outcomes for capitellum fracture with LCL injury.

Study	Cases (primary LCL injury)	Cases (LCL release)	Intervention	Pronation and supination	Active ROM	MEPS	EI	Complication
Giannicola et al. (14)	4/15	0/15	Anchors and/or transosseous sutures	Full	13°–140°	98 (75–100)	1	None
Zhang et al. (10)	4/28	—	Ligament repair	—	112°(60°–150°)	92.5 (62–100)	0	None
Dubberley et al. (13)	11/28	—	Fragment fixation (6), drill-holes and a locking suture technique (1), ligament repair (4)	Loss of 4°	19°–138°	91 (65–100)	—	None
Chang et al. (12)	0/9	9/9	Bone tunnel or fix to the plate	—	15°–135°	Not report	0	None
Ashwood et al. (7)	7/26	3/26	Suture anchors	Loss of over 10°	14.1°–128.8°	81.3 (65–100)	0	Pain (2), ulna nerve injury (2), superficial wound infection (4)
Ruchelsman et al. (20)	1/16	—	Reattached with #2 FiberWire and drill-holes	Full	123°	92	0	None
McKee et al. (18)	—/5	3/5	Suture through drill-holes	Full	15°–141°	—	0	Pain (2)
Tarallo et al. (24)	10/24	—	Transosseous suture (7), lateral screw (3)	Full	ROM 113.1°	92	1	None
Tarallo et al. (23)	4/8	—	Reinserted with transosseous sutures	Full	20°–125°	—	1	Heterotopic ossification (1), CRPS (1), LCL disruption (1)

ROM, range of motion; MEPS, Mayo elbow performance score; EI, elbow instability; CRPS, complex regional pain syndrome.

For cases with ligament injuries, the MEPS is often around 92, with one study as low as 81.5. In this study, suture anchors were used for repairing the LCL, while other studies adopted transosseous tunnel suture. This suggests that transosseous tunnel suture for LCL repair may help improve elbow joint function. For the repair of MCL injuries, this study used anchor repair, while other studies also included adjunctive use of hinged elbow external fixators, indicating that hinged elbow external fixators may bring benefits to the elbow joint.

A retrospective study by Giuseppe Giannicola et al. (14) reported that the proportion of associated MCL injury can be as high as 60% (9/15), while 4 suffered the lesion of LCL. The LCL were reinserted with anchors and/or transosseous sutures and fixed with a hinged external fixator for 6 weeks. However, the MCL injury was not specially surgically repaired primarily. In their opinion, the MCL injury may lead to valgus instability of the elbow. The mean range of elbow movement was 13° to 140°. Pronation and supination were full in all patients and no patients complained of pain. The average MEPS was 98 and no complications occurred.

A prospective study by Neil Ashwood et al. (7) recruited 26 patients with capitellum fracture. Four patients had MCL tear and seven patients had a complex LCL injury. All ligamentous injuries were reconstructed with suture anchors primarily to provide stability of the elbow and allow early active mobilization. Patients were followed for 46 months. Five complained pain on activity, 2 pain at rest and 19 no pain. The average range of elbow movement was 14.1° to 128.8°. The average MEPS was 81.3. The results were excellent in 9 patients, good in 9, and fair in 8. Eight complications, including pain (2), ulna nerve injury (2), superficial wound infection (4) were reported related to injury or surgery.

A retrospective analysis by Zhang et al. (10) included 28 patients with the capitellum fracture. Two patients had a MCL injury and 4 patients were associated with LCL injury. The LCL injury was repaired primarily while the MCL not. If the instability existed, the elbow was fixed with hinged elbow fixator. Patients were followed for 28.5 months. The average MEPS was 92.5. No patients complained pain and no elbow instability occurred.

A retrospective study by Dubberley et al. (13) included 28 patients with the capitellum fracture. Among these patients 2 had MCL injury and 11 had LCL injury. The LCL injury was repaired primarily by drill-holes and a locking suture technique. One patient with MCL injury was treated with a hinged brace and the other one treated with ligament repair. The patients were followed for 56 months and the average MEPS was 91.

Chang et al. (12) reported 9 patients with capitellum fracture. Primary ligamentous injury was not reported. However, the combined olecranon osteotomy and LUCL release approach were adopted. The LUCL was repaired by bone tunnel or fix to the plate to restore lateral stability. No cases of elbow instability or avascular necrosis occurred.

A retrospective study by Ruchelsman et al. (8, 20) included 16 patients, and only one patient suffered LCL injury. The LCL injury was repaired with #2 Fiber Wire and drill-holes. The MEPS was 92. Pronation and supination were the same as contralateral side. No elbow instability and no complications were reported.

Another study by McKee et al. (18) included 5 patients. None reported primary LCL injury, while 3 need the LCL elevated for enhanced exposure. LCL injury was repaired through drill holes. The pronation and supination were full. Two patients complained pain postoperatively. No elbow instability or other complications occurred.

Tarallo et al. published two reports (23, 24) on the capitellum fracture with ligament injury. A total of 32 patients were included. Lateral ligament injury was reported in 14 patients and reinserted with transosseous sutures or lateral screws. Full pronation and supination and satisfactory range of movements were achieved. Heterotopic ossification, pain, LCL disruption and elbow instability were found in on case respectively.

Several studies (19, 26) mentioned that the LCL origin was reflected distally to enhance exposure, but it must be repaired to restore elbow stability. However, the cases of LCL release were not reported.

## 4 Discussion

In this study, a total of 15 studies with 220 patients were finally included. MCL injury was reported in 4 while LCL in 9 studies. Two reports repaired MCL primarily and LCL reconstruction was performed in all studies. Of the included patients, 59 were male and 136 were female. The incidence was higher in female. The reason may be that women with osteoporosis are more likely to fracture during falls. Most of the included studies (13, 14, 16, 18, 19, 21) are consistent with this situation.

Capitellum fractures account for 1% of the elbow injuries and 6% of the distal humeral fractures. The associated injuries of capitellum fracture included elbow dislocation, ligamentous injury, fracture of distal humeral, radius and ulnar. The combined injury of capitellum fracture may have a negative impact on the functional outcomes of elbow (27). In the earlier literature, the incidence of humeral capitellum fracture with MCL injury was 5%-17% (27, 28). A study by Dubberley (13) in 2006 reported that the incidence of humeral capitellum fracture combined with ligament injury was as high as 39%. He believed that the prevalence of collateral ligament injury has been seriously underrated. In many reports, partial or complete ligament injuries were not routinely suspected or looked for during the management of these fractures (13). Another retrospective study (29) reported 67.2% (43/64) patients were associated with the capitellum injury in patients with a posterolateral dislocation of the elbow. In posterolateral dislocation of the elbow, MCL shows various degrees of injury, while the LCL ruptures are mostly complete. A high incidence of 61% of combined MCL injuries and capitellum fractures was reported by Johansson (30).

The treatment of LCL ligaments is closely related to the surgical approach. As an important component of the LCL, the LUCL is usually released in olecranon osteotomy to enhance exposure. Thus, during these procedures, the reconstruction of LCL is often performed to preserve the integrity of anatomic structure. In another case, when LUCL release is not performed but combined with LCL injury, the lateral approach enables the lateral structure fixation.Thus, the LCL is reconstructed when the primary LCL injury is confirmed.

Acute repair of collateral ligament injury/tear should be performed to ensure sufficient elbow stability to facilitate early mobilization (7, 31). This indicates that the concomitant ligament injury should be repaired primarily. However, other scholars hold different views. The anterior bundle of the medial collateral ligament is the most important structure to resist valgus stress. If the intra-articular fracture and ligament injury have been repaired or reconstructed, repair of the MCL is unnecessary (32).

For the management of MCL, the elbow stability is one of the most critical factors. Tenderness on the medial side of the elbow often indicates injury of the MCL (33). During the operation, elbow valgus was performed to check the stability of elbow and diagnose the MCL injury. In another study, four patients had a dislocation of elbow among the 30 patients with capitellum fracture. Half of the elbow had a redislocation after the surgery.

We suggest that the MCL should be explored and sutured primarily in the following cases. Firstly, when capitellum fracture combined with elbow dislocation, MCL should be repaired primarily. Besides, elbow instability still exists by intraoperative examination after LCL repair and fracture fixation. Finally, symptoms of ulnar nerve may still exist after reduction of elbow dislocation, which requires simultaneous exploration and release of ulnar nerve. The same incision can be used for exploration and repair of ligaments and nerves. This approach facilitates early mobilization and improves postoperative elbow stability. If the above terms are not met, MCL injury may require treatment in a hinged brace (33).

We conducted this study based on the published literature. Inevitably, there will be some shortcomings. Firstly, the information about ligament injury was not reported and unclear in some studies, which lead to the bias of the conclusion. Moreover, the extent of the damage, injury or rupture, was not specified in most reports. The curative effect and the complications depend on many factors, such as the severity of injury, the skill of the surgeon, and postoperative rehabilitation. The included literature is mostly retrospective or prospective clinical reports, without a control group. Clinical heterogeneity cannot be ignored, and there is no data for quantitative comparative analysis. This clinical heterogeneity may lead to biased results.

## 5 Conclusion

For capitellum fractures with ligament injuries, primary repair of LCL should be performed when combined by LCL injury or LUCL release is performed during surgery. The MCL may require primary reconstruction or treatment in a hinged brace.
